# Six-month blood pressure and glucose control among HIV-infected adults with elevated blood pressure and hyperglycemia in northern Tanzania: A prospective observational study

**DOI:** 10.1371/journal.pone.0285472

**Published:** 2023-05-08

**Authors:** Francis M. Sakita, Sainikitha Prattipati, Jordan Chick, Linda P. Samu, Amedeus V. Maro, Lauren Coaxum, Sophie W. Galson, Dorothy Samuel, Alexander T. Limkakeng, Paige R. O’Leary, Kajiru G. Kilonzo, Nathan M. Thielman, Gloria Temu, Julian T. Hertz

**Affiliations:** 1 Kilimanjaro Christian Medical University College, Moshi, Tanzania; 2 Kilimanjaro Christian Medical Center, Moshi, Tanzania; 3 Duke Global Health Institute, Duke University, Durham, North Carolina, United States of America; 4 Department of Emergency Medicine, Duke University School of Medicine, Durham, North Carolina, United States of America; 5 Health Department, Moshi Municipal Council, Moshi, Tanzania; 6 Majengo Health Centre, Moshi, Tanzania; 7 Department of Medicine, Duke University School of Medicine, Durham, North Carolina, United States of America; Elizabeth Glaser Pediatric AIDS Foundation, UNITED REPUBLIC OF TANZANIA

## Abstract

**Background:**

People with HIV in sub-Saharan Africa are increasingly developing age-related comorbidities. The purpose of this prospective observational study was to describe 6-month outcomes among Tanzanians with HIV and elevated blood pressure or hyperglycemia under current care pathways.

**Methods:**

Adults presenting for routine HIV care were enrolled and underwent blood pressure and blood glucose measurements. Participants with abnormal blood pressure or glucose were referred for further care, as per current guidelines. Participants’ blood pressure and point-of-care glucose were re-evaluated during their 6-month follow-up visit. Elevated blood pressure was defined as systolic ≥140 mmHg or diastolic ≥90 mmHg. Hyperglycemia was defined as fasting glucose ≥126 mg/dl or random glucose ≥200 mg/dl. An electrocardiogram was obtained at enrollment and at follow-up. Interim myocardial infarction and interim myocardial ischemia were defined as new pathologic Q waves and new T-wave inversions, respectively.

**Results:**

Of 500 participants, 155 had elevated blood pressure and 17 had hyperglycemia at enrolment. At 6-month follow-up, 7 (4.6%) of 155 participants with elevated blood pressure reported current use of an anti-hypertensive medication, 100 (66.2%) had persistent elevated blood pressure, 12 (7.9%) developed interim myocardial infarction, and 13 (8.6%) developed interim myocardial ischemia. Among 17 participants with hyperglycemia, 9 (56%) had persistent hyperglycemia at 6 months and 2 (12.5%) reported current use of an anti-hyperglycemic medication.

**Conclusions:**

Interventions are needed to improve non-communicable disease care pathways among Tanzanians with HIV.

## Introduction

Globally, hypertension and diabetes mellitus are leading risk factors for cardiovascular diseases (CVDs) such as stroke and myocardial infarction [1, 2]. Human Immunodeficiency Virus (HIV) is an independent risk factor for CVD [3, 4], and therefore people with HIV (PWH) who also have hypertension or diabetes are at particularly heightened risk for cardiovascular complications [5]. In sub-Saharan Africa, where the burden of HIV is greatest, advances in HIV care have resulted in substantially longer life expectancies among PWH [6]. As PWH in SSA live longer, they are increasingly developing age-related comorbidities, such as hypertension and diabetes [7, 8]. The growing burden of hypertension and diabetes among PWH in SSA presents a significant public health threat and portends a looming surge of CVDs among this high-risk population.

Preliminary data from across SSA suggests that the prevalence of hypertension and diabetes among PWH is already substantial. Small, cross-sectional studies from Malawi, Tanzania, South Africa, Ethiopia, and Kenya have reported hypertension prevalence of 13–29% and diabetes prevalence of 6–18% among adults engaged in HIV care [8–15]. Of particular concern, these preliminary studies also found that large proportions of patients with hypertension and diabetes were unaware of their condition and were not currently taking any anti-hypertensive or anti-hyperglycemic therapy. A recent study among PWH in Malawi, for example, found that 60% of adults with hypertension and 83% of those with diabetes had never been diagnosed with these conditions [8]. Multiple recent studies have investigated the feasibility of integrating HIV and non-communicable disease care, but integrated care models are not currently widespread in SSA [16]. In Tanzania, for example, current care guidelines call for siloed care between HIV and other chronic conditions [17]; therefore, PWH in Tanzania who are noted by their HIV provider to have elevated blood pressure or hyperglycemia would be referred to a separate facility for further management under current care processes. Little is known about the effectiveness of this partitioned referral system.

Moreover, despite a growing recognition of the substantial burden of poorly controlled noncommunicable comorbidities among PWH in SSA, few prospective studies report longitudinal outcomes of PWH with hypertension or diabetes. Indeed, most of the data describing noncommunicable comorbidities among PWH in the region have come from cross-sectional studies that do not report prospective outcomes, such as medication uptake after diagnosis, subsequent healthcare utilization, CVD incidence, or mortality. To address this knowledge gap, the purpose of this study was to describe six-month prospective outcomes including disease control, medication use, and healthcare utilization among Tanzanian adults with HIV and elevated blood pressure or hyperglycemia under current care models. Our primary aim was to determine the proportion of PWH who, after referral for further care for hyperglycemia or elevated blood pressure within the current siloed system, had persistent hyperglycemia or elevated blood pressure six months later. Since noncommunicable disease screening and care is not currently part of the national HIV treatment protocols in Tanzania, understanding healthcare utilization and outcomes among this population could have important implications for policy-making and clinical care guidelines [17].

## Materials and methods

### Study setting

This prospective observational cohort study was conducted in the Kilimanjaro Region of Tanzania, where the local prevalence of HIV, hypertension, and diabetes mellitus among the general adult population is 3%, 28%, and 6%, respectively [18–20]. Participant enrolment was conducted at the Majengo Care and Treatment Centre (MCTC), a government-funded outpatient HIV clinic that cares for approximately 1200 adults (800 women and 400 men aged ≥ 18 years). In general patients can establish care at MCTC either through external referral or self-referral. MCTC is located in an urban setting within the city of Moshi and serves clients both from within the city and from surrounding rural districts. At the time of this study, HIV-related care, such as antiretroviral therapy and HIV-related laboratory testing, was free to all MCTC patients, but noncommunicable disease screening and care was not provided as part of routine care. Under current protocols, MCTC patients attend follow-up appointments every 1 to 6 months, depending on how well-controlled their HIV infection is. MCTC operates three days per week (Monday, Wednesday, and Thursday), and recruitment was conducted during all clinic days. Clinical care at MCTC is typically provided by two clinical officers or physicians. Daily patient volumes at MCTC vary, but the clinic typically sees approximately 15–20 patients per day.

### Participant recruitment

Any adult (age ≥ 18 years) presenting to MCTC for HIV care was eligible for enrolment; there were no exclusion criteria. Trained research assistants approached consecutive adult MCTC patients to offer enrolment, which was conducted over a six-month period between September 1st, 2020, and March 1st, 2021. Although all MCTC patients consenting to participation were enrolled in the parent study [15, 21], only those who were found to have elevated blood pressure or hyperglycemia at time of enrolment were included in the present analysis, as detailed below.

### Study procedures

Initial study procedures have been described in detail elsewhere and are summarized here [15, 21, 22]. Enrolment and data collection were conducted by three research assistants, each with over a decade of experience conducting clinical research. Research assistants had either a bachelor’s degree or diploma in clinical medicine and underwent two weeks of dedicated training on study procedures prior to study commencement. Research assistants were not members of the MCTC staff. At time of enrolment, research assistants administered a standardized health questionnaire in Swahili to all participants [21]. This questionnaire, based on the World Health Organization (WHO) STEPS survey for noncommunicable disease [23], elicited sociodemographic information as well as information about personal medical history, medication use, and lifestyle behaviors [23]. The study questionnaire was typically administered while the patient was waiting to see the MCTC provider. Participant weight and height were also measured by the research assistants at time of enrolment. Information about HIV care history, including time since diagnosis, antiretroviral therapy, most recent CD4 count, and most recent HIV viral load, were collected directly from participants’ medical records.

Under current care protocols, HIV viral load and CD4 count are typically performed at least annually for MCTC patients in the MCTC lab. In some cases, no CD4 count or HIV viral load from within the past year were available in the medical record, typically because a patient had only recently established care at MCTC or because they had missed their appointment for bloodwork. In these cases where no CD4 count or HIV viral load were available in the medical record, a blood sample was collected by a research clinical officer for these assays during the patient’s six-month follow-up visit (described below). When CD4 count and viral load testing were performed by the study team, these assays were conducted at the Kilimanjaro Christian Research Institute Biotechnology Laboratory, using the BD FACSCount (Beckton Dickinson and Company, Franklin Lakes, New Jersey) and Abbott m2000 rt (Abbott Laboratories, Abbott Park, Illinois), respectively.

At enrolment, a resting twelve-lead electrocardiogram (ECG) was also obtained for all participants using the tablet-based PADECG (Edan Instruments, Shenzhen, China). At time of initial enrolment, the research assistants measured participant blood pressure, following American Heart Association/American College of Cardiology guidelines: blood pressure was measured with participant seated and arm at heart level, using an appropriately sized cuff, after participants had rested for about five minutes [24]. The Beurer BM40 automatic blood pressure monitor (Beurer, Ulm, Germany), was used for all blood pressure measurements. Research assistants also obtained a blood glucose level from all participants at time of enrolment, using a point-of-care glucometer (GlucoPlus Blood Glucose Monitoring System, GlucoPlus, Montreal, Canada). Participants were also asked whether they had consumed anything other than water on day of enrolment to ascertain whether the measured glucose result represented a random or fasting glucose level. Any participant with an elevated blood pressure or hyperglycemia (as defined below) was informed of the abnormal result and referred by MCTC staff to an adjacent general medical clinic, as per usual care; an MCTC nurse and physician was also immediately informed if any participant was noted to have elevated blood pressure or hyperglycemia for purposes of notification and referral. The general medical clinic to which patients were referred is a public facility staffed by physicians and clinical officers directly adjacent to MCTC. All clinical data obtained during the course of the study was shared with the clinical team at MCTC.

### Six-month follow-up

Six months after initial enrolment, participants were asked to return to MCTC for a follow-up study visit. Although no compensation was provided to participants at initial enrolment, reimbursement for transportation was provided (5000 Tanzanian shillings or approximately 2 USD) during the follow-up visit. Follow-up study visits were scheduled to coincide with routine clinic return visits to minimize any inconvenience for participants. Follow-up visits were conducted from March 1^st^ 2021 though September 1^st^ 2021. Follow-up surveys and data collection were performed by the same team of research assistants. At follow-up, participants underwent repeat blood pressure measurement, point-of-care glucose assay, and resting 12-lead ECG testing. They also completed a survey about new medication use and any interim unscheduled healthcare visits for chest pain, shortness of breath, hypertension, or diabetes. Participants were also asked if they were interested in more education about noncommunicable diseases. Finally, participants were asked if they would prefer to receive their noncommunicable disease care at their HIV clinic or in another facility. Participants with elevated blood pressure or hyperglycemia at follow-up were again referred to the adjacent medical clinic for further care.

### ECG interpretation

All ECGs were interpreted by two independent physician adjudicators, trained in either cardiology or emergency medicine. Adjudicators were blinded to all clinical data other than age and sex. Adjudicators were asked to determine presence of prior myocardial infarction and myocardial ischemia according to Fourth Universal Definition of Myocardial Infarction guidelines: prior myocardial infarction was defined as pathologic Q waves in ≥2 contiguous leads and myocardial ischemia was defined as T-wave inversions or ST-segment depressions in ≥2 contiguous leads [25]. In cases of disagreement, a third physician adjudicator served as the tiebreaker.

### Study definitions

Elevated blood pressure was defined as measured systolic blood pressure ≥140 mmHg or measured diastolic blood pressure ≥90 mmHg, corresponding to the threshold for Stage II Hypertension as per American Heart Association/American College of Cardiology guidelines [24]. Notably, these guidelines call for two separate blood pressure readings on two separate occasions to diagnose hypertension; due to the compressed timeline of this pilot study, only a single blood pressure reading was used to define elevated blood pressure in this study. Hyperglycemia was defined as fasting blood glucose ≥ 126 mg/dl or random blood glucose ≥ 200 mg/dl, as per American Diabetes Association guidelines [26]. Virologic suppression was defined as most recent HIV RNA viral load < 200 copies/ml. Sedentary lifestyle was defined as participant self-report of <150 minutes of moderately vigorous exercise per week and active lifestyle was defined by self-report of ≥150 minutes of moderately vigorous exercise per week, in accordance with WHO guidelines [27]. Medical co-morbidities, tobacco use, alcohol use, and fruit and vegetable consumption were also defined by participant self-report. Medication use at enrolment and follow-up was determined by participant self-report. Cases of interim myocardial infarction were defined as cases where pathologic Q waves consistent with prior MI were found on the 6-month follow-up ECG but not the initial ECG at enrolment. Similarly, cases of interim myocardial ischemia were defined as cases where pathologic T-wave inversions or ST-segment depressions consistent with myocardial ischemia were found on the 6-month follow-up ECG but not the initial ECG at enrolment. The primary outcomes of the study were blood pressure or glycemic control, defined as BP<140/90 or fasting glucose <126 or random glucose <200.

### Statistical approach

This was a purely descriptive study. For the present study, only participants meeting criteria for elevated blood pressure or hyperglycemia at initial enrolment were included in the analysis. All statistical analyses were performed in the R Suite [28]. Descriptive statistics were used to describe participant characteristics, blood pressure control, and glycemic control at baseline and six-month follow-up. Continuous variables are presented as medians and interquartile ranges (IQR) and categorical variables are presented as proportions. Body mass index (BMI) was calculated directly from measured weight and height. The sample size calculations for the parent study have been previously described [22].

### Ethics

All participants provided written informed consent prior to enrolment. This study received approval from ethical review committees at the Tanzania National Institute for Medical Research, Kilimanjaro Christian Medical Centre, and Duke Health.

## Results

**Fig 1:**
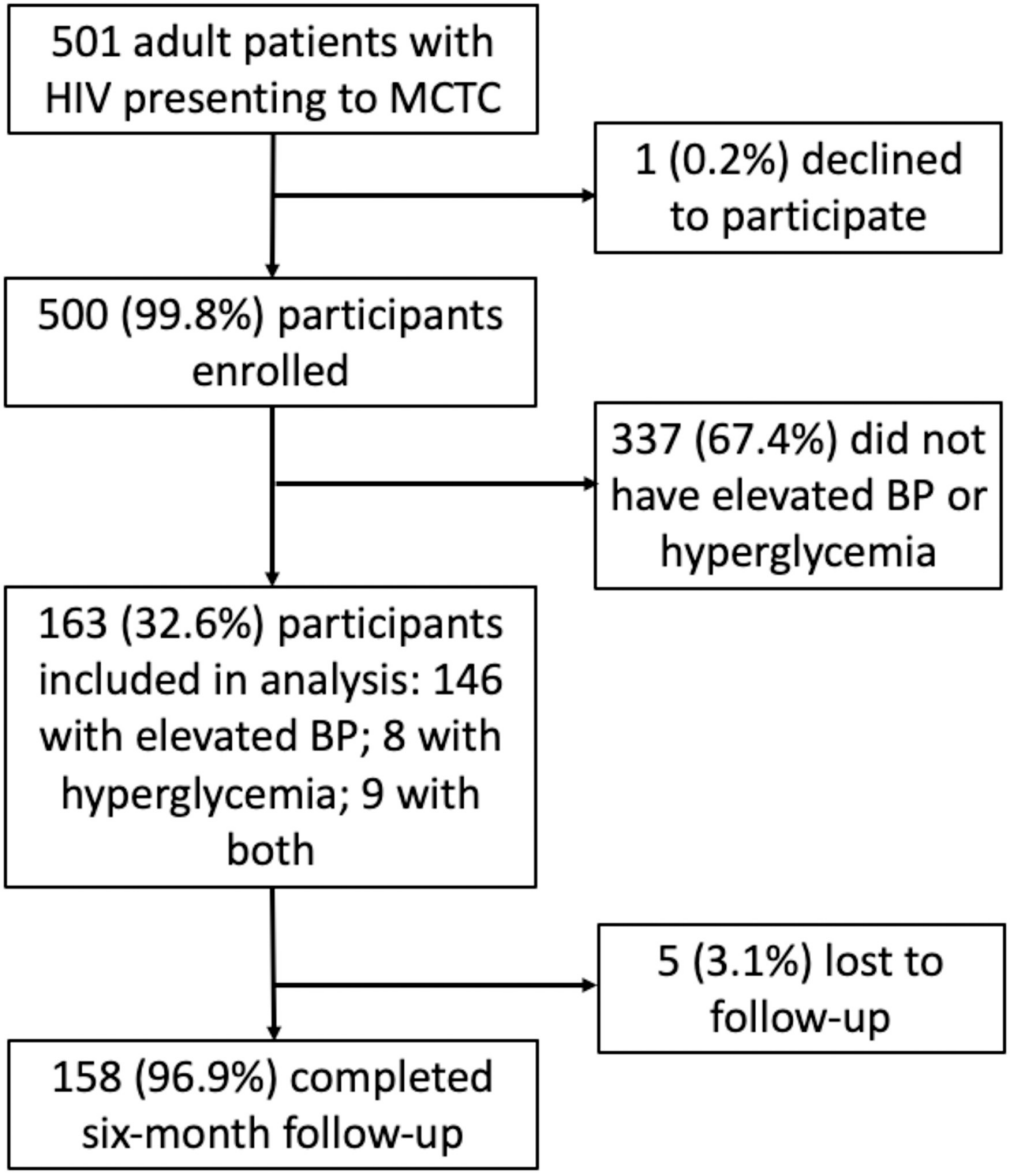
Study participant flow diagram, from recruitment through follow-up.

Of 501 MCTC patients approached for study inclusion, 500 (99.8%) consented to participate and were enrolled. Of these, 163 participants were included in the present analysis, including 146 (89.6%) participants with elevated blood pressure alone, 8 (4.9%) participants with hyperglycemia alone, and 9 (5.5%) participants with both elevated blood pressure and hyperglycemia (Fig 1). This resulted in N = 155 participants with baseline elevated blood pressure and N = 17 participants with baseline hyperglycemia.

Of note, 18 enrolled patients (3.6%) reported a history of hypertension but had well-controlled blood pressure (<140/90 mmHg) at enrolment and 4 (0.8%) participants reported a history of diabetes but did not have hyperglycemia at enrolment. These participants were excluded from the present analysis and are described elsewhere [15].

The baseline characteristics of participants with elevated blood pressure and hyperglycemia are summarized in Tables 1 and 2, respectively. Approximately three-quarters of participants with elevated blood pressure or hyperglycemia were female, consistent with the general MCTC clinic patient population. The median (IQR) age of participants with elevated blood pressure was 50 (44, 57) years, and the median (IQR) age of participants with hyperglycemia was 52 (46, 57) years. Few participants reported having health insurance. Of participants with elevated blood pressure, 39 (25.2%) reported a known history of hypertension and 3 (1.9%) reported current use of any anti-hypertensive medication. Of participants with hyperglycemia, 5 (29%) reported a known history of diabetes mellitus, and 2 (12%) reported current use of any anti-hyperglycemic medication.

**Table 1:** Baseline characteristics of HIV-infected adults with elevated blood pressure, northern Tanzania, 2020–2021 (N = 155).

Continuous variables	median	(IQR)
Age, years	50	(44, 57)
Body mass index, kg/m^2^	27.6	(23.7, 31.0)
Systolic blood pressure, mmHg	151	(143, 167)
Diastolic blood pressure, mmHg	94	(89, 99)
Categorical variables	n	(%)
Sex		
Male	40	(25.8%)
Female	115	(74.2%)
Highest formal education level		
None	9	(5.8%)
Primary school	116	(74.8%)
Secondary school	23	(14.8%)
University	7	(4.5%)
Health insurance		
Has health insurance	17	(11.0%)
Does not have health insurance	138	(89.0%)
Tobacco use		
Current tobacco use	6	(3.9%)
Does not use tobacco products	149	(96.1%)
Alcohol use		
Current alcohol use	77	(49.7%)
Does not drink alcohol	78	(50.3%)
Vegetable consumption		
Eats vegetables daily	36	(23.2%)
Does not eat vegetables daily	119	(76.8%)
Fruit consumption		
Eats fruits daily	31	(20.0%)
Does not eat fruits daily	124	(80.0%)
Physical activity		
Active lifestyle	104	(67.1%)
Sedentary lifestyle	51	(32.9%)
Known history of hypertension		
Self-reported history of hypertension	39	(25.2%)
No self-reported history of hypertension	116	(74.8%)
Anti-hypertensive medication use		
Current use of anti-hypertensive medication	3	(1.9%)
No current use	152	(98.1%)
Hypertension type		
Isolated systolic hypertension (≥140 mmHg)	44	(28.4%)
Isolated diastolic hypertension (≥90 mmHg)	25	(16.1%)
Combined systolic and diastolic hypertension	86	(55.5%)
Current anti-retroviral therapy		
TDF/lamivudine/dolutegravir	146	(94.2%)
TDF/lamivudine/efavirenz	4	(2.6%)
Other	5	(3.2%)

TDF: Tenofovir disoproxil fumarate

**Table 2:** Baseline characteristics of HIV-infected adults with hyperglycemia, northern Tanzania, 2020–2021 (N = 17).

Continuous variables	median	(IQR)
Age, years	52	(46, 57)
Body mass index, kg/m^2^	26.7	(24.6, 30.5)
Fasting glucose, mg/dl (N = 8)	2447	(195, 259)
Random glucose, mg/dl (N = 9)	304	(266, 335)
Categorical variables	n	(%)
Sex		
Male	3	(18%)
Female	14	(82%)
Highest formal education level		
None	1	(6%)
Primary school	13	(76%)
Secondary school	3	(18%)
Health insurance		
Has health insurance	2	(12%)
Does not have health insurance	15	(88%)
Tobacco use		
Current tobacco use	0	(0%)
Does not use tobacco products	17	(100%)
Alcohol use		
Current alcohol use	10	(59%)
Does not drink alcohol	7	(41%)
Vegetable consumption		
Eats vegetables daily	6	(35%)
Does not eat vegetables daily	11	(65%)
Fruit consumption		
Eats fruits daily	6	(35%)
Does not eat fruits daily	11	(65%)
Physical activity		
Active lifestyle	9	(53%)
Sedentary lifestyle	8	(47%)
Known history of diabetes		
Self-reported history of diabetes	5	(29%)
No self-reported history of diabetes	12	(71%)
Anti-hyperglycemic medication use		
Current use of anti-hyperglycemic medicine	2	(12%)
No current use	15	(88%)
Current anti-retroviral therapy		
TDF/lamivudine/dolutegravir	13	(76.5%)
TDF/lamivudine/efavirenz	2	(11.8%)
Other	2	(11.8%)

TDF: Tenofovir disoproxil fumarate

**Fig 2:**
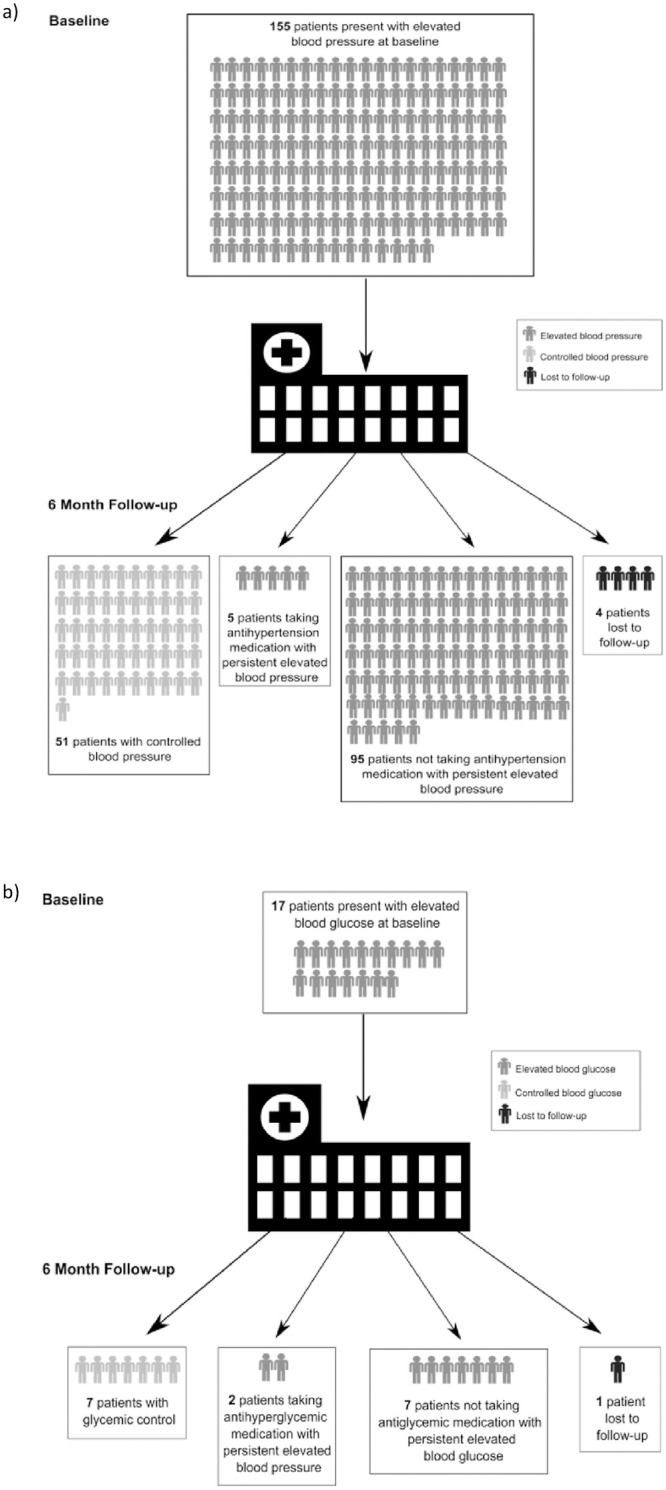
6–month outcomes among patients a) with elevated blood pressure and b) with hyperglycemia presenting to MCTC for HIV care in northern Tanzania.

Six months after enrolment, 5 (3.1%) participants were lost to follow-up, including 4 participants with elevated blood pressure and one participant with hyperglycemia. This resulted in 151 participants with baseline elevated blood pressure completing six-month follow-up and 16 participants with baseline hyperglycemia completing six-month follow-up. The six-month outcomes of participants with elevated blood pressure are presented in Table 3. Among 151 participants with baseline elevated blood pressure completing six-month follow-up, 100 (66.2%) participants had persistently elevated blood pressure, and 7 (4.6%) reported current use of an anti-hypertensive medication (Fig 2a). In the intervening six months, 28 (18.5%) participants reported an unscheduled healthcare encounter for chest pain, shortness of breath, or hypertension, 12 (7.9%) developed interval ECG findings of prior myocardial infarction, and 13 (8.6%) developed interval ECG findings of myocardial ischemia. The vast majority of participants with elevated blood pressure wanted more education about noncommunicable disease (n = 149, 98.7%) and preferred to receive their hypertension care at their HIV clinic (n = 114, 75.5%). Agreement among physician adjudicators regarding the presence of ischemia was strong (95.2% agreement, κ = 0.818).

**Table 3:** Six-month outcomes of HIV-infected adults with elevated blood pressure (N = 151^a^) or with hyperglycemia (N = 16^a^), northern Tanzania, 2021.

Health Status	Six-month outcome	n	(%)
HIV-infected adults with elevated blood pressure (N = 151)	Persistently elevated blood pressure	100	(66.2)
	Unscheduled healthcare encounter for chest pain, shortness of breath, or hypertension	28	(18.5)
	Current use of anti-hypertensive medication	7	(4.6)
	Interim ECG changes		
	Interim myocardial infarction on ECG	12	(7.9)
	Interim myocardial ischemia on ECG	13	(8.6)
	Would like more education about noncommunicable disease	149	(98.7)
	Would prefer to receive noncommunicable disease care at the HIV clinic	114	(75.5)
HIV-infected adults with hyperglycemia (N = 16)	Persistent hyperglycemia	9	(56)
	Unscheduled healthcare encounter for chest pain, shortness of breath, or hyperglycemia	4	(25)
	Current use of anti-hyperglycemic medication	2	(13)
	Interim ECG changes		
	Interim myocardial infarction on ECG	0	(0)
	Interim myocardial ischemia on ECG	0	(0)
	Would like more education about noncommunicable disease	15	(94)
	Would prefer to receive noncommunicable disease care at the HIV clinic	11	(69)

ECG: electrocardiogram

^a^ Denominators exclude 4 participants with baseline elevated blood pressure who were lost to follow-up and 1 participant with baseline hyperglycemia who was lost to follow-up. Nine participants with both baseline elevated blood pressure and baseline hyperglycemia were included in both sections of Table 3.

The six-month outcomes of participants with hyperglycemia are presented in Table 3. Among 16 participants with hyperglycemia completing six-month follow-up, 9 (56%) participants had persistent hyperglycemia and 2 (13%) reported current use of an anti-hyperglycemic medication (Fig 2b). Four (25%) reported an unscheduled healthcare encounter for chest pain, shortness of breath, or hyperglycemia over the prior six months. No participant had interval development of ECG findings of myocardial infarction or myocardial ischemia.

## Discussion

To our knowledge this is one of the first studies to report prospective outcomes of PWH with elevated blood pressure or hyperglycemia in SSA. As advancements in HIV care have led to increased life-expectancy for PWH in SSA, there has been an increase in the burden of age-related, NCDs among this population [6–8, 29]. In our study, few patients with elevated blood pressure or hyperglycemia reported a history of hypertension or diabetes diagnosis at time of enrolment, likely due to low levels of NCD screening and awareness in this population [21, 27]. At 6-month follow-up, the majority of participants did not achieve adequate blood pressure or glycemic control, and uptake of anti-hypertensive or anti-hyperglycemic medications during the follow-up period was low. Of further concern, we observed a large number of NCD-related healthcare visits over the six-month follow-up period and ischemic ECG changes among 10% of participants with elevated blood pressure. Overall, these findings demonstrate the need to improve current care pathways for NCDs among PWH in Tanzania.

At 6-month follow-up, most participants with elevated blood pressure or hyperglycemia did not achieve guideline-recommended blood pressure or glycemic targets. These low rates of NCD control are likely due, at least in part, to the observed low uptake of anti-hypertensive or anti-hyperglycemic medications among participants. Further study is needed to explore reasons for low uptake of these medications, since all participants were referred to an adjacent general practitioner if they were found to have elevated blood pressure or hyperglycemia at initial enrolment. Recent qualitative studies have identified multiple barriers to anti-hypertensive and anti-hyperglycemic therapies among PWH in Tanzania, including fear, lack of understanding, and cost [30, 31]. In contrast to antiretroviral therapies, anti-hypertensive and anti-hyperglycemic medications are not provided free-of-charge in Tanzania. As the vast majority of our participants did not report health insurance coverage, out-of-pocket medication costs may have been a barrier to NCD medication uptake in our study [32, 33]. Additional study is needed to further identify and explore barriers to hypertension and diabetes control among PWH in Tanzania. Regardless, the fact that so many participants in our study who were referred to a separate facility for management of elevated blood pressure or hyperglycemia had persistently uncontrolled blood pressure and hyperglycemia six months later demonstrates that existing siloed care pathways are insufficient to provide optimal control of NCD comorbidities in this population. Currently, there are scant comparative data describing prospective NCD outcomes among PWH in SSA. In the SEARCH trial in Uganda, which evaluated an integrated HIV and hypertension clinic model, 48% of PWH and hypertension achieved blood pressure control at 1 year [34], more than the 34% of participants who achieved blood pressure control at 6 months in our study. Additional study is needed to describe prospective NCD outcomes among PWH in SSA and develop interventions to improve NCD control in this population.

Beyond low rates of NCD control, we also observed frequent healthcare utilization in our cohort: roughly one in five participants reported an unscheduled visit to a healthcare facility for chest pain, shortness of breath, or hyperglycemia. This finding suggests that there may be substantial cost benefits, at the individual and system levels, to improving NCD control among this high-risk population. Of further concern, we found that more than one in ten participants with elevated blood pressure developed new ECG changes suggestive of myocardial infarction or myocardial ischemia within 6 months of enrolment. Although ECG findings of Q waves and T-wave inversions are non-specific and may indicate a wide array of pathologies including cardiomyopathies, structural heart disease, and coronary ischemia [35, 36], the presence of these interim ischemic ECG changes is notable and suggests that end-organ damage may be occurring among Tanzanians with HIV and poorly-controlled hypertension over a relatively short six-month period. Indeed, ischemic ECG findings like Q waves are strongly predictive of future myocardial infarction and death [37], and so these findings underscore the need for better NCD control to prevent future cardiovascular events in this high-risk population.

There was a strong desire for more NCD education among our participants, and most expressed a preference for receiving NCD care at the HIV clinic. In recent years, many public health experts have advocated for an integrated healthcare model in SSA: providing NCD and HIV care at one clinic [16, 37–39]. The integrated care model has been piloted in a variety of SSA settings and has been shown to be both feasible and well accepted by patients [16, 40–43]. Although rigorous cost-effectiveness data remains scarce, recent studies in Uganda and Tanzania found that integration of HIV and NCD care may be substantially more cost-effective than siloed care [44–46]. However, the effect of integration on NCD control remains unclear, with many studies demonstrating minimal or no increase in the proportion of patients achieving blood pressure control when compared to usual care [16, 40–42, 47]. In Tanzania, where integrated NCD care is not routinely provided alongside HIV care, additional study is needed to identify best practices for maximizing NCD control among PWH in a cost-effective fashion [48].

This study had multiple limitations. Because we only enrolled patients who were already engaged in HIV care, our findings may not be generalizable to PWH who are not engaged in care or are unaware of their HIV status. Patients not engaged in care are likely to have even lower rates of NCD control. Secondly, we relied on random glucose measurements to identify patients with potential diabetes mellitus due to cost restraints. Hemoglobin A1c levels would likely have provided a more accurate measure of the prevalence and control of diabetes mellitus among participants, although hemoglobin A1c may be less accurate in those with HIV [49]. Furthermore, we measured blood pressure on only two occasions, separated by 6 months. Continuous ambulatory blood pressure monitoring would have provided a more comprehensive description of blood pressure control among participants. Because blood pressure is highly variable and may be transiently elevated due to white coat hypertension or other factors, we may have included participants without true underlying hypertension [50]. The term “elevated blood pressure” was used to describe our participants to indicate that they had not met the formal diagnostic criteria for hypertension. Moreover, the use of a single blood pressure screening to identify patients with elevated blood pressure likely resulted in exclusion of patients with hypertension who for various reasons had transiently depressed blood pressure at time of screening; screening for elevated blood pressure over multiple occasions would likely have identified additional participants with elevated blood pressure. We also relied on participant self-report for medication use, which could have led to under- or over-reporting of medication uptake due to recall bias and social desirability bias. Additionally, we excluded a handful of patients who reported a history of hypertension or diabetes but did not have elevated blood pressure or hyperglycemia at enrolment. Although there were few such patients, exclusion of these patients likely resulted in an under-estimation of the effectiveness of current care pathways in controlling comorbid hypertension and diabetes. Another limitation of our study is that we did not observe care of referred patients at the outside medical clinic or investigate participants’ reasons for not taking antihypertensive or antihyperglycemic medications at follow-up; such data would have shed further light on reasons for poor blood pressure and glycemic control in our study population. Finally, we used ECG changes including new T-wave inversions, new ST-segment depressions, and new pathologic Q waves as a measure of interim myocardial ischemia or infarction, but these findings are non-specific and may be seen in other conditions such as pulmonary embolism and non-ischemic cardiomyopathies [48, 51, 52].

In conclusion, among Tanzanians with HIV and comorbid elevated blood pressure and hyperglycemia at a single center, most had not achieved adequate blood pressure or blood glucose control, and most were not taking any anti-hypertensive or anti-hyperglycemic medications six months after initial screening. Importantly, because we do not know whether these participants received a formal diagnosis of hypertension or diabetes or whether they were prescribed any medications, the reasons for their persistently elevated blood pressure or their persistent hyperglycemia are unclear and warrant further exploration. There was a strong desire among patients for more NCD education, and there is an urgent need to develop locally tailored interventions to improve NCD care pathways among PWH in Tanzania.

## Supporting information

S1 Dataset (XLSX)

S1 File Inclusivity in global research. (DOCX)
